# Using the Equivalent Fiber Approach in Two-Scale Modeling of the Elastic Behavior of Carbon Nanotube/Epoxy Nanocomposite

**DOI:** 10.3390/nano8090696

**Published:** 2018-09-06

**Authors:** Mahdi Javadinejad, Mohammad Mashayekhi, Mehdi Karevan, Homayoun Hadavinia

**Affiliations:** 1 Mechanical Engineering Group, Pardis College, Isfahan University of Technology, Isfahan 84156-83111, Iran; mmjavadinejad@gmail.com; 2 Department of Mechanical Engineering, Isfahan University of Technology, Isfahan 84156-83111, Iran; mkarevan@cc.iut.ac.ir; 3 Department of Mechanical Engineering, Kingston University, London SW15 3DW, UK

**Keywords:** carbon nanotube, interface, cohesive element, equivalent fiber, CNT agglomeration

## Abstract

In this study, the mechanical behavior of epoxy/carbon nanotubes (CNTs) nanocomposite is predicated by a two-scale modeling approach. At the nanoscale, a CNT, the interface between the CNT and the matrix and a layer of the matrix around the CNT are modeled and the elastic behavior of the equivalent fiber (EF) has been identified. The CNT/epoxy interface behavior is modeled by the Park–Paulino–Roesler (PPR) potential. At the microscale, the EFs are embedded in the matrix with the extracted elastic properties from the nanoscale model. The random pattern has been used for the dispersing of EFs in the representative volume element (RVE). The effect of CNTs agglomeration in the epoxy matrix has also been investigated at the micro level. The Young’s modulus of the nanocomposite was extracted from simulation of the RVE. CNT/epoxy nanocomposites at four different volume fractions were manufactured and the modeling results were validated by tensile tests. The results of the numerical models are in good agreement with the experiments and micromechanics theory, and by considering agglomeration of CNT in the model, the modeling results match with the experiments.

## 1. Introduction

The use of polymer matrix composites in recent years has been growing dramatically. The recent development of nanotechnology and incorporation of nanoparticles in polymer matrix resulted in new generation of nanocomposites with enhanced properties. The result is increased use of polymer nanocomposites in various industries such as aeronautics, marine, automotive, medicine, and sports equipment [1,2]. Epoxy is a thermoset polymer with relatively light weight, low cost, high strength, and high Young’s modulus which can be easily combined with other materials, in spite of limitations such as low thermal conductivity and poor flame resistance. These advantages make the epoxy an excellent choice to manufacture all kinds of polymer nanocomposites [3,4,5,6].

For establishing connections between network structure and various physical and mechanical properties such as glass transition, modulus in the glassy state and development of residual stresses series of epoxy–amine networks of well-controlled architecture were studied [7]. In addition, the yielding behavior of epoxy–amine resins was investigated by a study of the stress–strain curves in compression mode, recorded at various temperatures and strain rates. Two types of antiplasticizer were examined, depending on whether they remain miscible to the network or give rise to nanoscale phase separation along network construction, lead to improved toughness [8].

Various nanoparticles are used for improvement of mechanical properties of epoxy such as carbon nanotubes (CNTs), graphite, graphene oxide, clay, silicon nitride, silica, and nitride [9]. Among these, CNTs/epoxy nanocomposite has extremely high mechanical properties [3,10,11]. It should be noted that the use of CNTs is not limited to the strengthening of the nanocomposites. Some other properties such as low weight, high thermal stability, high electrical conductivity, and high chemical resistance make CNTs a proper candidate for enhancing nanocomposite properties [12]. Enhancing the electrical conductivity of polymers has boosted their growth in electronics and for lightning strike protection in aerospace applications.

The prediction of the mechanical properties of nanocomposites by numerical methods is a very suitable solution for reducing the extent of experimental work and as a result reducing the production costs. The methods used for the prediction of mechanical properties of nanocomposites can be classified as (i) theories based on continuum mechanics, (ii) atomistic modeling, and (iii) numerical continuum mechanics [13]. In this study, the finite element method (FEM) based on the numerical continuum mechanics approach has been used for modeling CNT/epoxy nanocomposite.

The experimental study of CNTs reinforced polymers is time consuming and incurs high cost. On the other hand, due to the hypotheses imposed on the micromechanical equations, it is not possible to use them to predict the mechanical properties of nanocomposites containing CNTs nanoparticles. On the other hand, computationally, the use of FEM is superior to the molecular dynamics in terms of solution time, but considering the interface as a continuum medium with homogeneous properties without modeling the interatomic potential of the van der Waals forces is not consistent with the actual behavior of the material.

Odegard et al. [14] introduced a method using a combination of molecular dynamics, a nanostructure, and a continuum mechanics theory to calculate the mechanical properties of nanocomposites. They showed that their method could be used to predict the mechanical properties of polymer nanocomposites with single-wall carbon nanotubes (SWCNTs) in a directional or random orientation. Wan et al. [15] calculated Young’s modulus of nanocomposites by considering an isotropic layer at the interface with its Young’s modulus a multiple of the polymer matrix Young’s modulus. Liu and Chen [16,17] modeled the cylindrical and cubic RVE of nanocomposite using the FEM, assuming prefect bond at the interfacial phase. They considered this volumetric element as reinforcement and used the rule-of-mixtures (ROM); then, the results were compared with the experiments showing that the mechanical properties of the volumetric element created by this method could be reliable. Using a cubic shaped RVE, Fereidoon et al. [18] created a 3D model of CNTs and polymers around them. In their model, CNTs were modeled with the beam elements, showing that the changes in Young’s modulus increases linearly with increasing the CNTs volume fraction.

Shokrieh and Rafiee [19] also obtained the Young’s modulus of the nanocomposite by using a FEM and modeling van der Waals forces at the interface by using the nonlinear spring elements at the nanoscale. Comparison of the results by rule-of-mixtures showed that this rule did not have the capability to predict the elastic properties of nanocomposites and had an approximation more than the actual values. Shojaie and Golestanian [20] considered a cubic RVE containing a CNT. They simulated the interface as a thin film with elastic properties and compared the results with those of micromechanics. The modeling was done by changing the mechanical properties of the interface region to reach perfect bond. The results are compared with those from rule-of-mixtures showed that the mechanical properties of the nanocomposite were significantly dependent on the surface strength of the interface. In a similar study, Joshi et al. [21] calculated Young’s modulus of the nanocomposite by the FEM using a hexagonal RVE. They studied the effect of changing the angle of the CNT on the Young’s modulus. They found that the Young’s modulus of the RVE was dependent on the angle of the CNTs; by increasing the angle of the CNT relative to the axis of loading, the Young’s modulus was decreased. Deng et al. [22] have shown experimentally that the effective elastic modulus of CNT as reinforcement of polymer is far less than the theoretical value. Using a simple tensile test on a nanocomposite film whose particles were polarized by the laser and comparison of the results with the rule-of-mixtures, the value of the effective elastic modulus of the nanoparticles was declared to be between 50% and 70% of the theoretical value.

Peng et al. [23] used a two-scale modeling to create a 2D FEM of nanocomposite with polymer nanoparticles. The interface was considered as an effective interface and according to the elliptical geometry of the nanoparticles, the effect of aspect ratio, radius of the nanoparticle, orientations, volume fraction, and clustering on the Young’s modulus of subcell and unit cell were investigated. The results were compared and validated with the results of Mori-Tanaka approach. Eventually, they showed that the elastic modulus increases with increasing the aspect ratio of nanoparticles. Also, they showed the thickness and properties of the interface, the shape of the nanoparticles, their orientation, and degree of clustering have strong influence on the elastic modulus of nanocomposite.

Zuberi and Esat [24] used FEM to model the interface by two perfect bond methods, using truss/spring elements. They found that the nanotube structure affected the properties of the RVE, and the Armchair structure causes a higher increase of properties than Chiral and Zigzag types. Subramanian et al. [25] simulated the traction–separation behavior at the interface in order to achieve force-displacement cohesive behavior by molecular dynamics. By extracting the result from different fracture modes, they developed some analytical equations based on the interface behavior. 

Previous studies indicate that the modeling of nanocomposites involves many requirements, and all of them add to the complexity of the modeling and the calculations become complicated and time-consuming. As a result, by selecting the more important and effective parameters, the computational time can be reduced to an acceptable level while still having high accuracy. The interface surface between CNTs and epoxy is one of the major parameters in nanocomposites modeling. Often, molecular dynamics simulation or its results are used to model the interface but it involves a high computational time [26,27]. In some studies, the interface is considered as perfect bond [16,17,18,28]; however, the interface between the nonfunctionalized CNTs and the surrounding resin in the normal state consists of weak van der Waals bonds. Some scholars have compensated for errors, due to van der Waals bonds at the interface, by considering their properties as a multiple of the properties of CNT and polymer [20,21].

In the present study, the Young’s modulus of CNT/epoxy nanocomposite has been evaluated at a multiscale level. The interface behavior is modeled at nanoscale as it is the most significant feature of nanocomposites as compared with the conventional composites. Using the EF technique at this scale can reduce computational costs in simulations at the larger scale. At the microscale, the EF is distributed as reinforcement with properties extracted from the RVE at nanoscale. Due to the fact that the EF outer layer and the matrix of the RVE are of the same material, the bond between them is considered to be perfect. Finally, the simulation results are compared with the experimental results as well as with the results from the Halpin–Tsai model [29,30].

## 2. Nanocomposite Modeling

From Marino’s point of view, important sources of error in simulating the behavior of nanocomposites can be attributed to interfaces, agglomeration, and morphology [13]. The modeling of the interface between resin and nanoparticles is one of the main issues in the present study. Given that the applied CNT in this study is nonfunctionalized, the behavior of the interface is based on van der Waals forces between the nanotubes and polymer molecules; it is defined by the Park–Paulino–Roesler (PPR) potential model and is implemented with user subroutine UEL in the ABAQUS finite element software. On a volumetric scale, the dispersion pattern for nanofibers is taken randomly.

### 2.1. Interface 

In conventional composites, the forces between the matrix and the fibers account for a small proportion of the interatomic forces in the structure of the composite. But in nanocomposites, where the surface-to-volume ratio is much higher, these forces are more important. Therefore, a model that can simulate the surface and the interatomic forces of the surface more precisely is appropriate [31]. In the current research, cohesive elements are used to model the interface behavior. The behavior of these elements is defined based on a nonlinear traction–separation relationship. From an atomistic perspective, failure occurs when the separation between atoms occurs.

**Figure 1:**
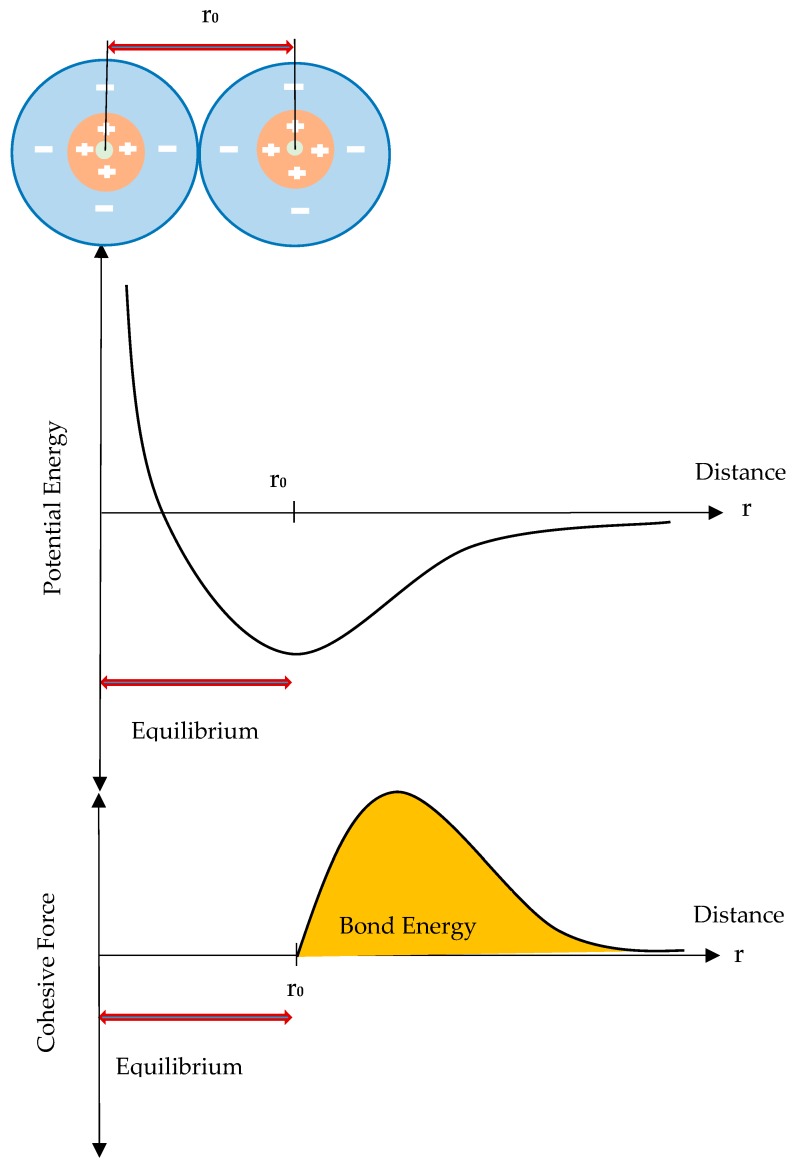
Variation of the potential energy and the cohesive force during separation of two atoms from each other.

Figure 1 shows a schematic of the behavior between the two atoms and the energy required for their separation [32]. Accordingly, the cohesive force between the atoms acts in a nonlinear manner in separation and the assumption of the linear behavior for this force is not correct.

Carbon nanotubes are cylindrical molecules consisting of a circular array of sp^2^ hybridized carbon atoms, which makes it impossible to form a covalent bond between the nanotube carbon atoms, and the bond with the surrounding polymer molecules can be achieved through weak van der Waals forces [25,33]. Given the fact that the energy contribution of van der Waals interactions in the intermediate phase is three times higher than the electrostatic bond energy, the electrostatic bonds are ignored compared to the van der Waals bonds [34]. The bond between the carbon atoms in the outermost layer of the untreated CNT and the matrix molecules on the interface is formed by van der Waals bonds, which can be defined by the Lennard–Jones (L–J) Potential function. The L–J Potential is the most commonly used expression for the attraction/repulsion properties of the interaction between atoms and neutral molecules, expressed by Equation (1) [31].

(1) $$V_{LJ}\left(r\right)=4\varepsilon \left[{\left(\frac{\sigma }{r}\right)}^{12}-{\left(\frac{\sigma }{r}\right)}^{6}\right]$$

*r* is the distance between the atoms, and *ε* and *σ* are the van der Waals parameters, which are 0.4492 kJ/mol and 0.34 nm for carbon atoms, respectively. The variation of the Lennard–Jones force in terms of the distance between the carbon atoms is shown in Equation (2) [35]:

(2) $$F_{LJ}\left(r\right)=-24\frac{\varepsilon }{\sigma }\left[2{\left(\frac{\sigma }{r}\right)}^{13}-{\left(\frac{\sigma }{r}\right)}^{7}\right]$$

**Figure 2:**
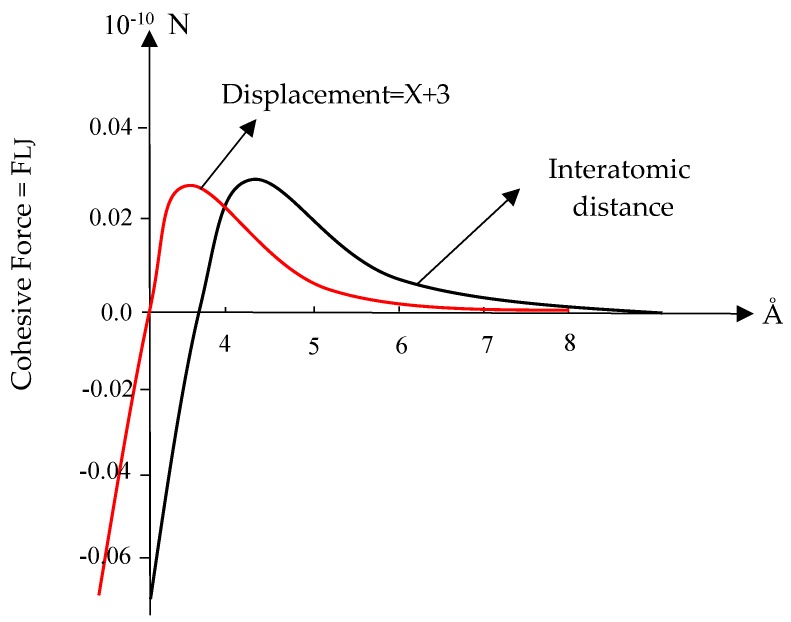
The van der Waals force versus interatomic distance and displacement curve.

The van der Waals force versus interatomic distance as well as force versus displacement are depicted in Figure 2 [36,37]. Jiang et al. [38], Tan et al. [39], and Zakeri et al. [36] are among the researchers used the L–J Potential to calculate the cohesive force at the interface between CNTs and polymer matrices.

The equilibrium distance in Equation (1) for carbon atoms is $r_{0}=3.8\;Å$, and for the calculation of forces in terms of displacement, we can rewrite Equation (2) in the form of Equation (3). In this equation, *x* is the amount of atomic displacement [30].

(3) $$F_{LJ}\left(r\right)=-24\frac{\varepsilon }{\sigma }\left[2{\left(\frac{\sigma }{x+3.8}\right)}^{13}-{\left(\frac{\sigma }{x+3.8}\right)}^{7}\right]$$

### 2.2. Potential Model 

Until now, there have been different equations for force-displacement between the adhesive surfaces, but none of them are capable of defining the nonlinear behavior on the adhesive surface completely. As a result, there is a difference between the actual behavior of the interface with these models. Park, Paulino, and Roesler (PPR) derived the relation between the potential sticky surfaces as presented in the Equation (4) [40].

(4) $$\begin{array}{l} \Psi (\Delta_{n},\Delta_{t})=\min(\phi_{n},\phi_{t})+\left[\Gamma_{n}{\left(1-\frac{\Delta_{n}}{\delta_{n}}\right)}^{\alpha }{\left(\frac{m}{\alpha }+\frac{\Delta_{n}}{\delta_{n}}\right)}^{m}+\langle \phi_{n}-\phi_{t}\rangle \right]\times \\ \left[\Gamma_{t}{\left(1-\frac{\left|\Delta_{t}\right|}{\delta_{t}}\right)}^{\beta }{\left(\frac{n}{\beta }+\frac{\left|\Delta_{t}\right|}{\delta_{t}}\right)}^{n}+\langle \phi_{t}-\phi_{n}\rangle \right] \end{array}$$

In Equation (4) *δ_n_*, *δ_t_*, Г*_n_*, and Г*_t_* are:

(5) $$\delta_{t}=\frac{\phi_{t}}{\tau_{\max}}\beta \lambda_{t}{\left(1-\lambda_{t}\right)}^{\beta -1}(\frac{\beta }{n}+1){\left(\frac{\beta }{n}\lambda_{t}+1\right)}^{n-1}$$

(6) $$\delta_{n}=\frac{\phi_{n}}{\sigma_{\max}}\alpha \lambda_{n}{\left(1-\lambda_{n}\right)}^{\alpha -1}(\frac{\alpha }{m}+1){\left(\frac{\alpha }{m}\lambda_{n}+1\right)}^{m-1}$$

(7) $$\Gamma_{n}={\left(-\phi_{n}\right)}^{\frac{\langle \phi_{n}-\phi_{t}\rangle }{\left(\phi_{n}-\phi_{t}\right)}}{\left(\frac{\alpha }{m}\right)}^{m},\Gamma_{t}={\left(-\phi_{t}\right)}^{\frac{\langle \phi_{t}-\phi_{n}\rangle }{\left(\phi_{t}-\phi_{n}\right)}}{\left(\frac{\beta }{n}\right)}^{m}$$

**Figure 3:**
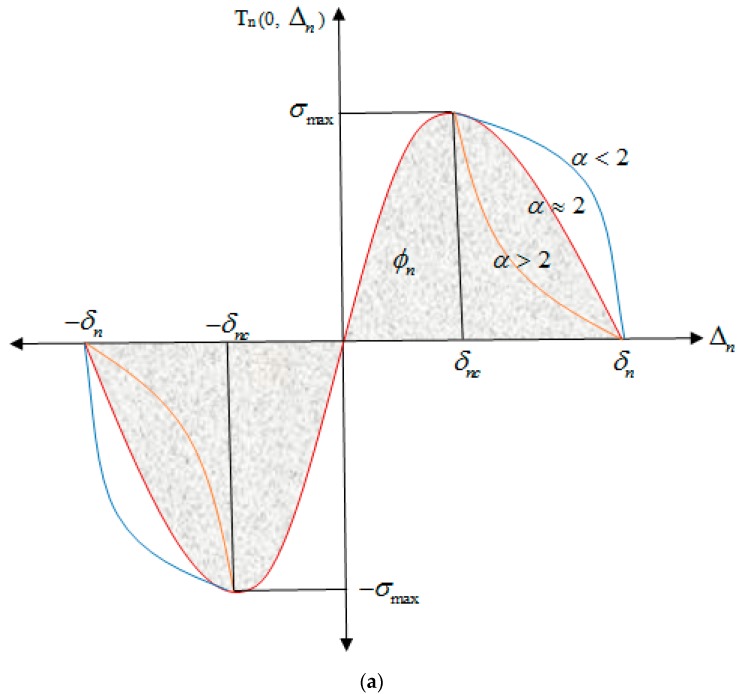

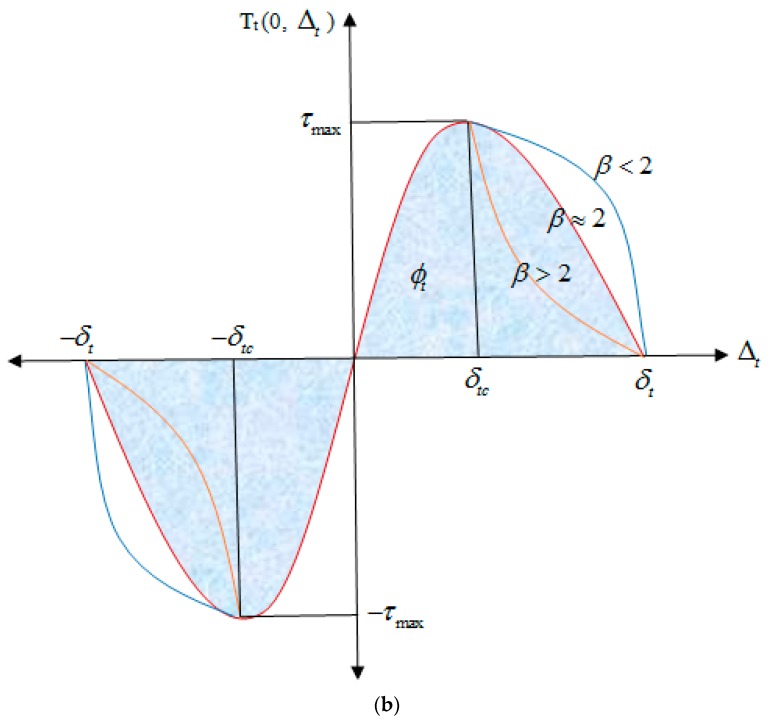
Traction–separation diagram in the (**a**) normal and (**b**) tangential mode [40,42].

There are eight input parameters in the PPR potential model: modes I and II fracture energies (*ϕ_n_*, *ϕ_t_*), normal and tangential cohesive strengths (*σ*_max_, *τ*_max_), normal and tangential shape parameters (*β*, *α*), and the initial slope indicators (*λ_t_*, *λ_n_*), according to Figure 3. The characteristic length scale parameters (*δ_n_*, *δ_t_*) is given by the Equations (5) and (6), as well as their relation with other parameters [40,41].

Appendix A describes how to calculate these parameters in the tangential mode [35]. If the energy values of the normal and tangential fracture are equal, then Equation (7) becomes the same as Equation (8). *m* and *n* are calculated by means of the Equation (9), based on the shape parameters in the PPR model (*α*, *β*) and the initial slope indicators, *λ_n_* and *λ_t_* which are defined by the Equation (10).

(8) $$\Gamma_{n}=-\phi {\left(\frac{\alpha }{m}\right)}^{m},\Gamma_{t}={\left(\frac{\beta }{n}\right)}^{n}$$

(9) $$m=\frac{\alpha (\alpha -1)\lambda_{n}^{2}}{\left(1-\alpha \lambda_{n}^{2}\right)},n=\frac{\beta (\beta -1)\lambda_{t}^{2}}{\left(1-\beta \lambda_{t}^{2}\right)}$$

(10) $$\lambda_{n}=\frac{\delta_{nc}}{\delta_{n}},\lambda_{t}=\frac{\delta_{tc}}{\delta_{t}}$$

**Figure 4:**
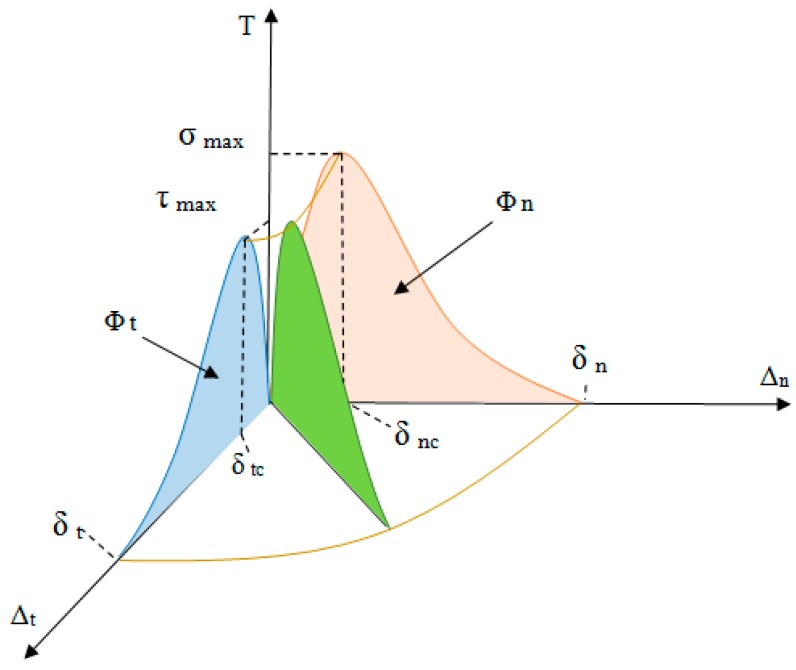
Normal and tangential mixed-mode response of the cohesive element in a three-dimensional state.

Figure 3 schematically shows the traction–separation diagram in the normal and tangential mode based on the PPR potential model [40]. In Figure 4, the traction–separation diagram is shown in the mixed mode. Since there is a good agreement between the Lennard–Jones variation diagram in Figure 2 and the PPR model in Figure 4, this potential can be used as the basis for defining the adhesive properties in the form of nonlinear elasticity at the interface, whose main parameters values are based on the L–J potential.

The strain energy of interface on a continuous surface can be calculated using Cauchy–Born rule as shown in Equation (11).

(11) $$\phi =\frac{\int V_{LJ}\left(r\right)dr}{S_{0}},(S_{0}=\frac{3\sqrt{3}}{4}r_{ij}^{2})$$

where *r_ij_* is the distance between carbon atoms [43,44]. Using this idea makes it possible to simulate the interface by considering its actual behavior. The potential model of the Equation (4) is implemented for use in the finite element software and coded in the Fortran [45]. The eight input parameters for PPR potential model are calculated by Equations (4)–(11), Figure 2, and Appendix A listed in Table 1.

### 2.3. Equivalent Fiber 

**Figure 5:**
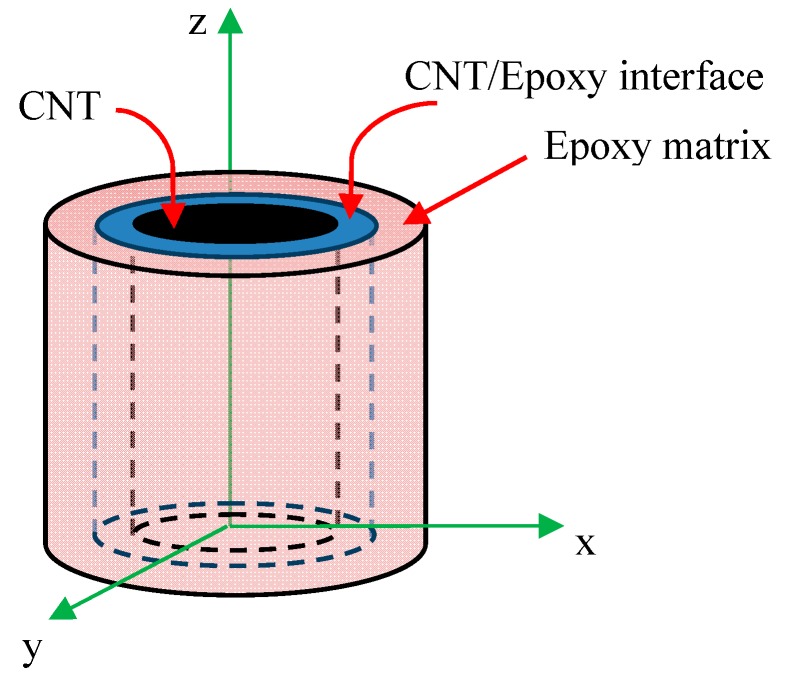
A schematic of the equivalent fiber model.

The use of EF technique is an efficient method for reducing the volume of computation. By using this method, the behavior of nanocomposites is similar to that of conventional composites [16,20,21,27]. It should be noted that the structure of the epoxy matrix at the vicinity of the CNT surface differs from the bulk epoxy due to the formation of an ultrathin epoxy layer at the CNT/epoxy interface. This ultrathin layer consists of highly packed crystalline epoxy monomers, which has different elastic properties in comparison with the amorphous bulk polymer [46,47]. It is shown that the radial distribution function (RDF) of epoxy atoms is zero at the radial distance of 0.56 nm and reaches its maximum value of 160 atoms/nm^3^ at the radial distance of 0.77 nm [48]. Then, it starts to fluctuate around an average value of 110 atoms/nm^3^. This result indicates that the value of the van der Waals equilibrium distance is ∼2.75 Å and the thickness of CNT/epoxy matrix interphase layer is ∼3.0 Å. The schematic of the equivalent fiber model is shown in Figure 5. The mechanical properties of the nanotubes are based on the material which is used in experimental tests, and is shown in Table 2 [49,50]. The multiwalled carbon nanotubes (MWCNTs) are made by the USNANO company, with an inside diameter of 5 to 10 nm, an outer diameter of 10 to 20 nm, and a length of 10 to 30 μm. In the modeling, the CNTs’ average internal diameter of 7.5 nm and the external diameter of 15 nm were assumed. KER828 epoxy with the Young’s modulus of 2.6 GPa, Poisson’s ratio of 0.3, and density of 1.16 g/cm³ was chosen. The resin Young’s modulus was obtained through tensile test according to the ASTM-D638 standard. Due to the high aspect ratio of MWCNT, the modeling of CNTs in the RVE was subjected to limitations. In order to create the finite element model, researchers have considered the aspect ratio between 6 and 70 [49].

## 3. Simulation of Nanocomposite Behavior

The high computational costs for atomic and molecular dynamics modeling of the behavior of nanocomposites at a nanoscale, as well as the limitations of these methods in higher scales, and the difference between the results of micromechanical theories and the experimental ones have all led researchers to consider alternative methods. In fact, the difference between atomic behaviors and continuum mechanics hypotheses is an obstacle to the use of these methods. The use of multiscale methods and the transfer of properties from smaller scale to a higher one with the aim of reducing the computational time have been considered in many studies employing the FEM and atomistic methods [27,35,37,48,51,52,53]. In this study, the multiscale approach is used for modeling atomic behavior at the interface. Finite element simulation is used at nanoscale and microscale for the prediction of the nanocomposite Young’s modulus. At the nanoscale, the EF was simulated and its mechanical properties were extracted and transferred to microscale model.

### 3.1. Equivalent Fiber Simulation

**Figure 6:**
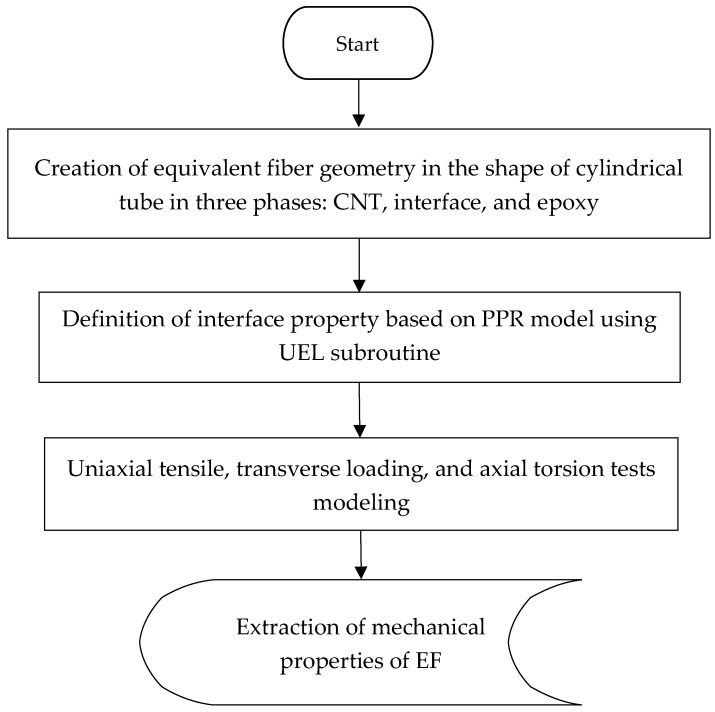
Modeling steps at the nanoscale.

In this section, the finite element is simulated according to Section 2.3. Since the ABAQUS software library does not include cohesive elements with the nonlinear elastic behavior, a user element subroutine (UEL) was written to define the interface behavior based on the PPR potential model. The input parameters of the model were extracted and applied, using the L–J atomic potential by using Equations (1)–(4), as shown in Figure 2. The finite element simulations of the uniaxial tensile, transverse loading, and axial torsion tests have been performed. From finite element analysis (FEA) results and Equations (12)–(17), the mechanical properties of the EF, including transverse and longitudinal Young’s modulus, the shear modulus, and Poisson’s ratio were extracted. Figure 6 illustrates the steps of the modeling at nanoscale.

**Figure 7:**
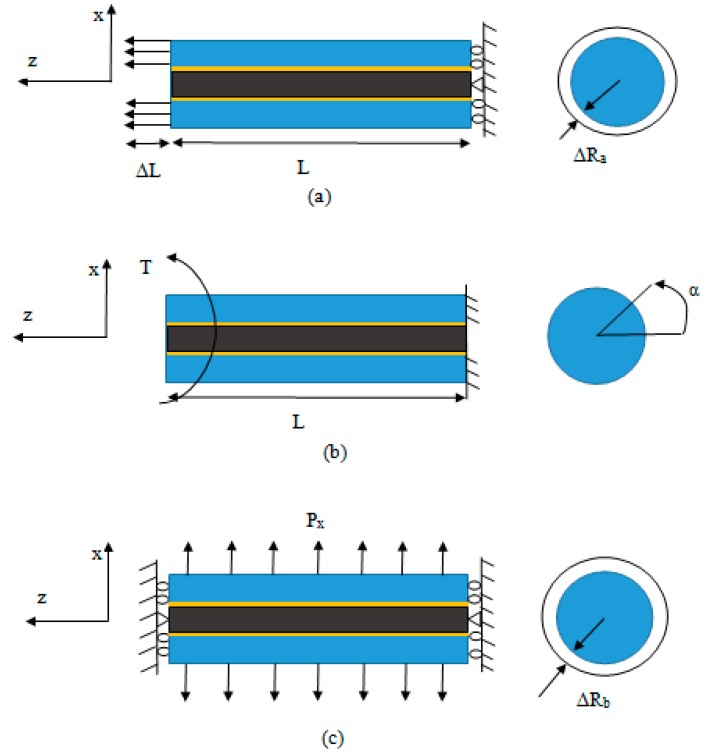
Performing (**a**) axial tensile; (**b**) torsional; and (**c**) transverse tests in simulation.

**Figure 8:**
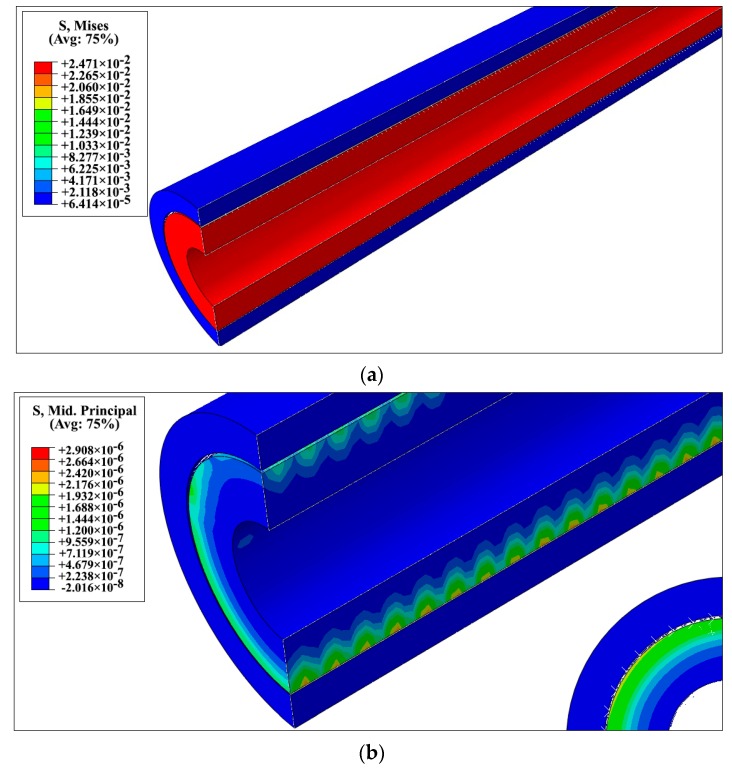
Results of finite element simulation of equivalent fiber (EF) under uniaxial loading (**a**) contour plots of von Mises stress (**b**) mid-principal stress distribution.

The EF was simulated in the uniaxial tensile loading as shown in Figure 7, and the reaction force at the support is computed and longitudinal Young’s modulus, $E_{z}$, has been extracted from Equation (12) and Poisson’s ratio, $\nu_{zx}$, from Equation (13). Moreover, in the transverse loading, the EF has been constrained at both ends along its axial direction to make a plane strain condition; then, Equation (14) was used for calculation of the transverse Young’s modulus. Also, by applying torsional loading, variation of the twist angle in the EF was computed and the shear modulus was found using Equation (15). By solving Equations (14) and (15), the Young’s modulus, *E_x_*, and Poisson’s ratio, *v_xy_*, can be obtained as shown in Equations (16) and (17). In this work, the study is focused on the effect of CNTs on the Young’s modulus of the epoxy in the elastic limit. For investigating the strength of the CNT reinforced epoxy nanocomposite at a microscale, the effect of interfacial failure and the local damage should be considered. The distributions of von Mises stress and mid principal stress of EF under uniaxial tensile loading are shown in Figure 8.

Calculation of the longitudinal Young’s modulus:

(12) $$E_{z}=\frac{P_{z}L}{A\Delta L}=\frac{\sigma }{\varepsilon }$$

Calculation of the Poisson’s ratio between the longitudinal axis and the transverse plane:

(13) $$\upsilon_{zx}=\frac{-\left(\frac{\Delta R_{a}}{R}\right)}{\left(\frac{\Delta L}{L}\right)}$$

Elastic equation for transverse direction:

(14) $$-\left(\frac{\upsilon_{xy}}{E_{x}}+\frac{\upsilon_{zx}{}^{2}}{E_{z}}\right)+\left(\frac{1}{E_{x}}-\frac{\upsilon_{zx}{}^{2}}{E_{z}}\right)=\frac{\Delta R_{b}}{P_{x}R}$$

Calculation of the shear modulus:

(15) $$\frac{TL}{\alpha J}=G_{xy}=\frac{E_{x}}{2(1+\upsilon_{xy})}$$

Young’s modulus, *E_x_*, and Poisson’s ratio, *v_xy_*, are calculated from Equations (16) and (17):

(16) $$E_{x}=E_{y}=\frac{2}{\frac{1}{2G_{xy}}+\frac{2\nu_{zx}^{2}}{E_{z}}+\frac{\Delta R_{b}}{P_{x}R}}$$

(17) $$\nu_{xy}=\frac{E_{x}}{2G_{xy}}-1=\frac{1}{G_{xy}(\frac{1}{2G_{xy}}+\frac{2\nu_{zx}^{2}}{E_{z}}+\frac{\Delta R_{b}}{P_{x}R})}-1$$

The actual length of the CNTs is based on the production process, the arrangement of atoms, and the number of walls, ranges from 100 nm to 15 μm. The dispersion of the EFs in the RVE with the actual length of the CNTs is awkward.

**Figure 9:**
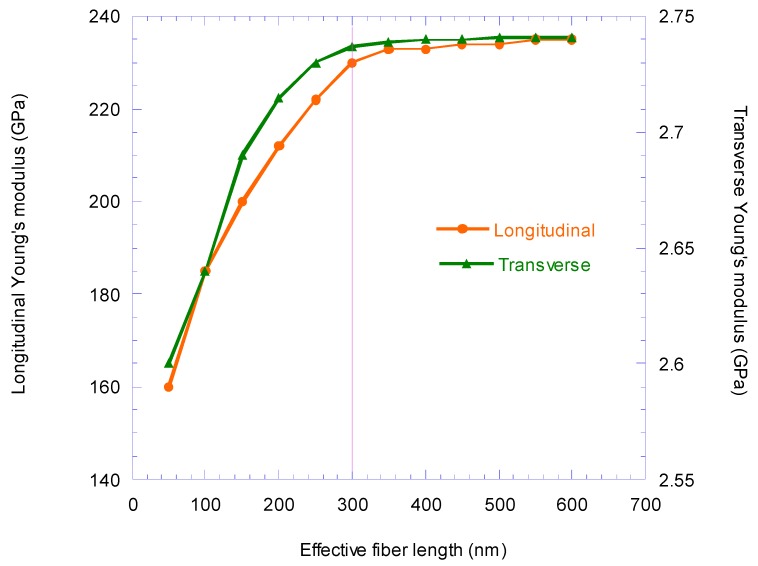
Variation in the longitudinal and transverse Young’s modulus with respect to the change in EF length.

Due to the aspect ratio of CNTs (~700), the equivalent fiber boundary conditions are defined to model an infinite length for the carbon nanotube. Figure 9 illustrates variation in the longitudinal and transverse Young’s modulus with respect to the change in EF length from 50 nm to 600 nm. This diagram shows that the longitudinal Young’s modulus does not change significantly for EF lengths around 300 nm, and for fibers length over 300 nm, the change in transverse Young’s modulus of EF changes is negligible; hence, in the modeling of EF its lengths was considered equal to 300 nm.

**Figure 10:**
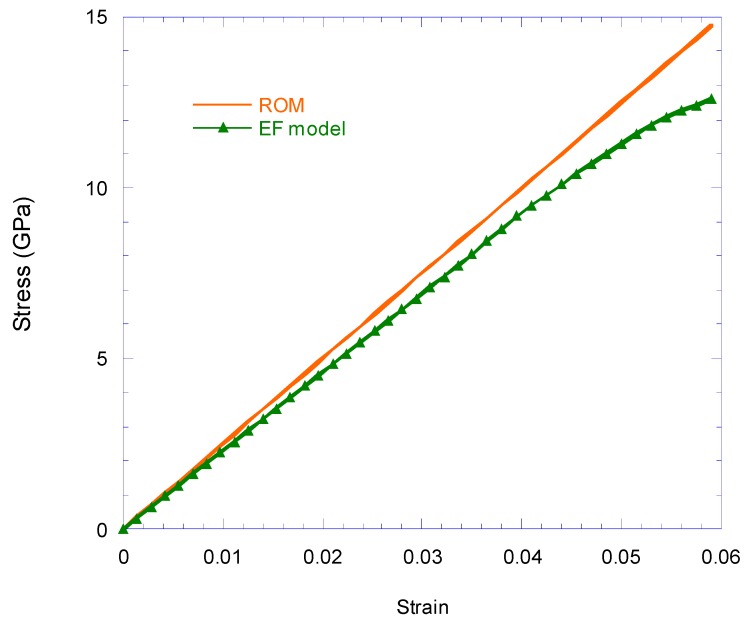
Comparison of longitudinal stress–strain behavior of the finite element analysis EF model and the rule-of-mixtures (ROM).

In Figure 10, the longitudinal stress–strain curve obtained from the FEM for EF is compared with the results of the rule-of-mixtures. The results show that up to the strain of 0.045 the behavior is linear. The results obtained for the mechanical properties of the EF also shows that the EF behavior is transversely isotropic, and this behavior was due to the behavior of the interface and the transversely isotropic nature of CNTs.

### 3.2. RVE Simulation

**Figure 11:**
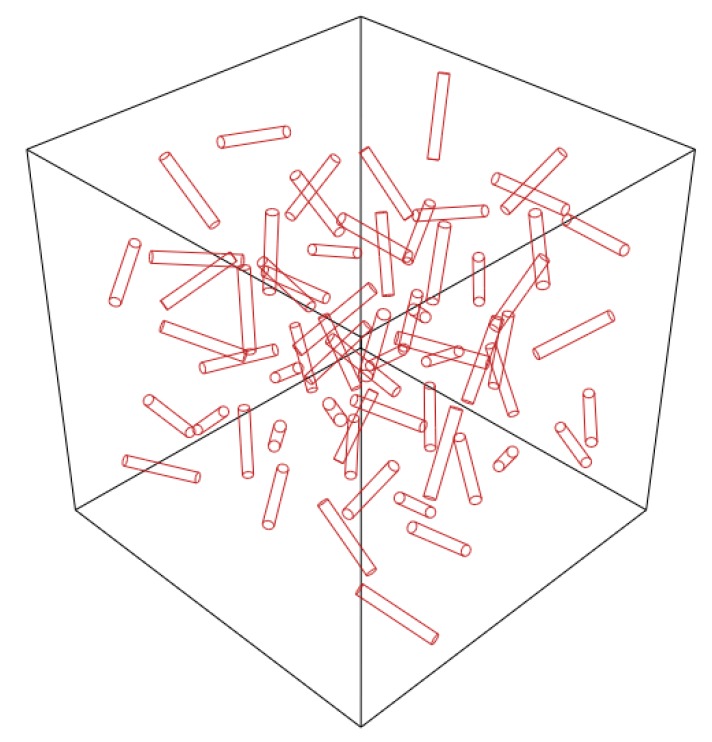
3D view of the representative volume element (RVE) with the volume of 1500 × 1500 × 1500 nm³ and the random dispersion of the equivalent fibers.

**Figure 12:**
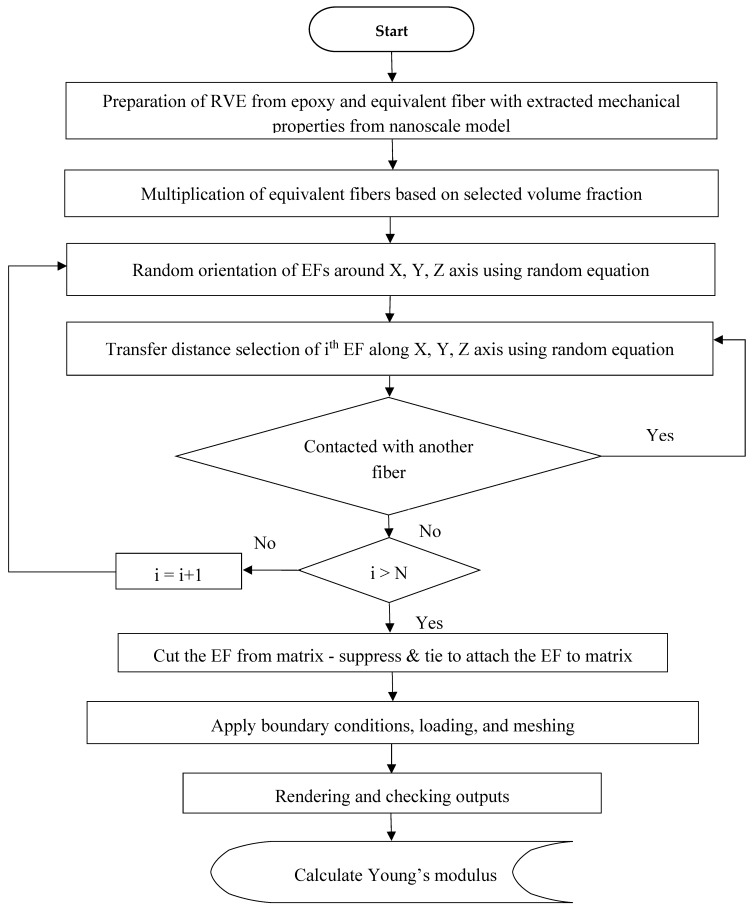
Flowchart of the RVE modeling at the microscale.

This study has been done for volume fractions of less than 1%, and dispersion is done by ultrasonic energy. Reducing the volume fraction of CNTs has a direct effect on the agglomeration [10]. The agglomeration of CNTs is ignored in this section and the effect of CNTs clustering by the FEM is investigated in the next section. In the simulation of the RVE, it is assumed that the bond between the EF and the matrix is a perfect bond and the position and angle of the reinforcement are chosen randomly. This model is implemented by the Python script in the ABAQUS, which allows the script to select and modify the properties and dimensions of the matrix and the reinforcement, and the values and properties of the EF are based on nanoscale outputs. Figure 11 shows the RVE with dimensions of (1500 × 1500 × 1500) nm^3^. The Young’s modulus of the RVE was calculated using Equation (18). In Figure 12 the algorithm of the RVE simulation is shown. The results of this model, as the full dispersion model, are presented in Section 5.

(18) $$E=\frac{PL}{A\Delta L}=\frac{P}{A\varepsilon }$$

### 3.3. Simulation of CNTs Agglomeration

**Figure 13:**
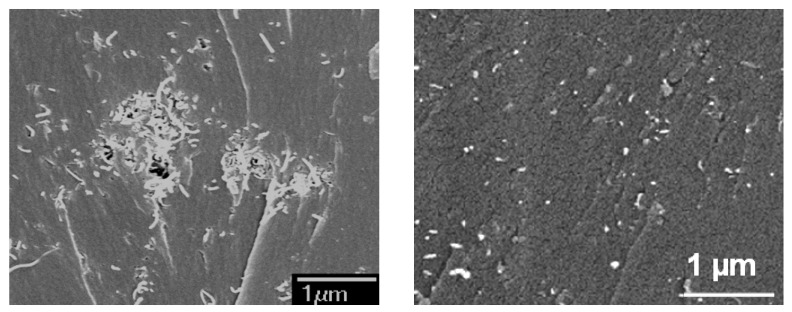
SEM images of fracture surface of epoxy containing 0.5 wt.% pristine carbon nanotubes (CNTs) (**left**) and amino-CNTs (**right**). Reproduced with permission from [54]. Elsevier, 2010.

Incorporation of CNTs can improve significantly the mechanical properties of epoxy at <3 wt% loading if they are properly dispersed and good interfacial bonding between the CNTs and polymer matrix is achieved. However, due to strong intermolecular van der Waals interactions, CNTs tend to form ropes or bundles. These bundles can aggregate further, forming entangled networks or agglomerates; hence deteriorating the mechanical properties of nanocomposite as contribution from individual CNTs cannot be optimally exploited. One of the methods for improving dispersion and interfacial adhesion of CNTs is amino-functionalization. Typically, carboxylic groups (–COOH) are produced by treating CNTs with high concentration acids, followed by further functionalization using amine molecules. Figure 13 compares dispersion of pristine CNT and aminofuntionalized-CNT in an epoxy resin [54,55,56]. Therefore, it is important to fully understand the effect of agglomeration of CNTs on mechanical behavior of nanocomposite.

**Figure 14:**
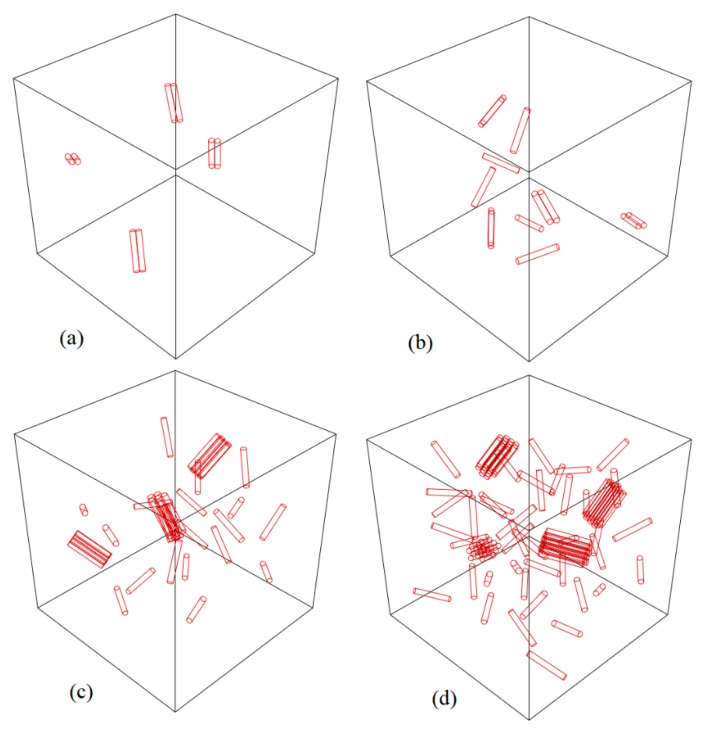
3D view of the RVE with clustering of CNTs in four colonies: (**a**) 0.18 wt%; (**b**) 0.36 wt%; (**c**) 0.9 wt%; (**d**) 1.8 wt% (half of the EFs are clustered at four random point).

In this section, the effect of agglomeration on the Young’s modulus of nanocomposite has been investigated by FEM. The RVE is divided into 64 sub cubes and half of the CNTs are clustered in 1, 2, and 4 colonies, respectively, and the rest are dispersed randomly in RVE. Figure 14 shows a 3D view of the RVE with four colonies in different volume fractions. For a volume fraction of 1%, which is most effective against agglomeration, the effect of the ratio of nanotubes aggregated to the whole nanotube in RVE is investigated in four states of 25%, 50%, 75%, and 100%. The results of these models, as the agglomeration model, are presented in Section 5.

## 4. Experimental Studies

Nanocomposite specimens were made and tested in accordance with the ASTM-D638 standard Type V. The production of the samples was done using multistage production instructions aimed at the highest degree of separation and dispersion. Silicon was used for molding, which is a thermosetting polymer group with good flexibility, allowing for the removal of specimens without any damage. Acetone was used as a solvent for better dispersion of MWCNTs in the epoxy. 

Table 3 shows the amount of each of the constituents’ materials for different volume fractions of CNTs.

**Figure 15:**
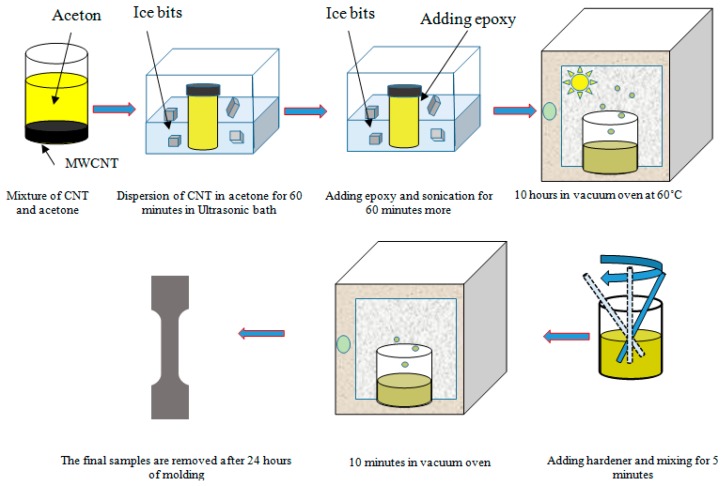
The production stages of CNT/epoxy nanocomposite.

**Figure 16:**
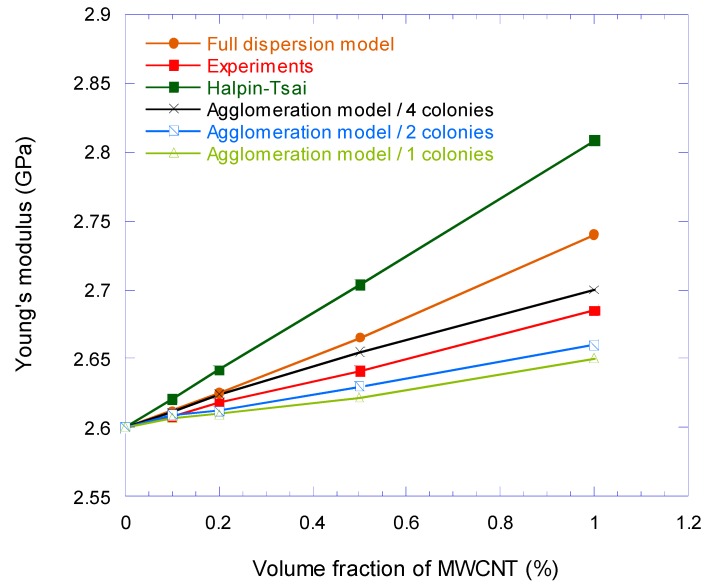
Variation of Young’s modulus of CNT/epoxy nanocomposite with respect to MWCNT volume fraction.

Figure 15 illustrates the process of nanocomposite specimens’ production. To make the specimen, the CNT was first mixed with acetone and placed in an ultrasonic bath for an hour. After that, the epoxy was added and placed in an ultrasonic bath for another hour. This process dispersed CNTs in the epoxy. In the next step, for the removal of acetone and air bubbles, the nanocomposite was placed in a vacuum oven for 10 h (to be sure, the mass of the mixture was measured after removal from the oven). Then, by adding the hardener, the mixture was slowly mixed for 5 min and placed in a vacuum oven for 10 min. At the end, the nanocomposite was poured into the dogbone mold and after 24 h, the specimens were ready for tensile testing. Tensile test was conducted according to the ASTM-D638V standard with the universal testing machine SANTAM-STM50. The values of the Young’s modulus obtained from this experiment were compared with the results of the simulation and the Halpin–Tsai equation in Figure 16.

## 5. Result and Discussion

According to the results shown in Figure 10, for EF length longer than 300 nm, the longitudinal and transverse EF Young’s modulus does not depend on its length. A longitudinal Young’s modulus of 230 GPa and transverse Young’s modulus of 2.74 GPa were obtained. Although the linear behavior was considered to be for CNTs and epoxy, a nonlinear behavior is exhibited at strains greater than 0.045 of the EF, which is similar to the nature of the van der Waals bonds at the interface.

The FEA simulation results are compared with Halpin–Tsai model defined in Equations (A5)–(A9) in Appendix B, along with the experimental results. The results of nanocomposite Young’s modulus form experiment, Halpin–Tsai and full dispersion FEA simulation together with agglomerated CNTs FEA models for 1, 2, and 4 colonies are compared in Figure 16. The results show that the values obtained from simulations are between the results from Halpin–Tsai equation and experiments and the agglomerated CNTs FEA model with four colonies is the closest to the experiment. This shows that the dispersion of CNTs in the epoxy was not perfect and there were some agglomerations.

**Figure 17:**
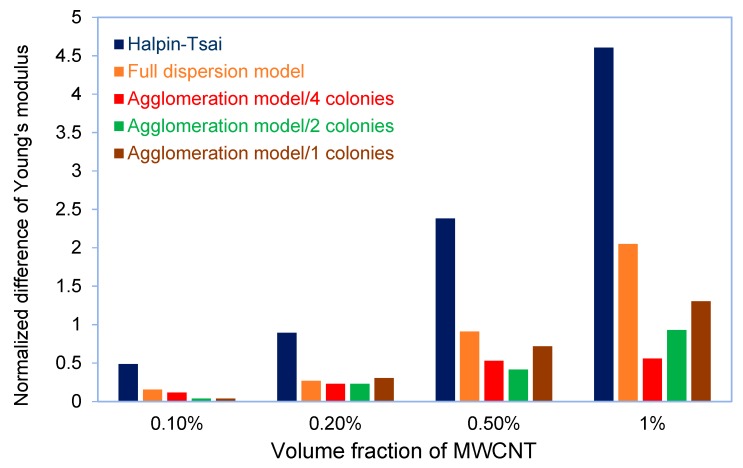
Comparison of the normalized difference of Young’s modulus from FEA modeling and Halpin–Tsai equation relative to the experimental results.

**Figure 18:**
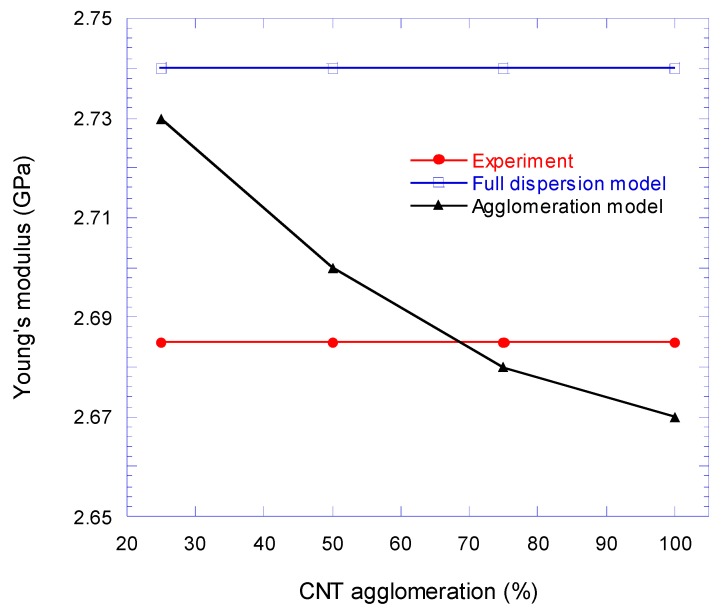
Effect of CNTs agglomeration on Young’s modulus of CNT/epoxy nanocomposite for four colonies model and 1 Vf.% CNT loading.

The full dispersion model showed that for MWCNTs loading at 1 Vf.%, the Young’s modulus of epoxy nanocomposite was increased by 5%. This number was 1.7% more than the experimental value, while the result from the Halpin–Tsai equation overestimated 4.6% the experimental value at the same volume fraction. If the results are compared with the rule-of-mixtures, the difference will be more than 20%. The proximity of the results of the FEA modeling with the experimental ones showed the effect of interface modeling on the elastic behavior and the Young’s modulus of the nanocomposite. In Figure 17, the normalized differences of Young’s modulus, which are derived from the FEA simulation and Halpin–Tsai equation, are compared with the experimental results. As can be seen in this figure, by increasing the amount of the CNT volume fraction, the difference between the experimental results and the simulation increases and the highest difference at the volume fraction of 1% is 2.05%. However, this deviation is approximately half less than the difference of Halpin–Tsai equation and the experiment. The augmentative growth of normalized difference between the full dispersion model and experiment by increasing the volume fraction of CNT is rooted in agglomeration at higher loading of the CNT nanoparticles. Figure 18. shows the effect of clustered CNTs percentage (in four colonies) in a 1% volume fraction on Young’s modulus. As can be seen, the increase in agglomerated CNTs volume fraction decreases the modulus of the nanocomposite.

Using the PPR potential-based cohesive model made the results of simulation approach the experimental results closely, which was very accurate in comparison with micromechanical theories. Hence, the nonlinear elastic model could be used to define the adhesive behavior between atomic surfaces, and simulate the proper pattern of interface behavior in the nanocomposites.

## 6. Conclusions

In this study a CNT/epoxy nanocomposite has been simulated to investigate the elastic behavior of the nanocomposite in tension. At the nanoscale, the interface has been modeled by nonlinear cohesive element. The atomistic behavior of Lennard–Jones is linked with the Park–Paulino–Roesler (PPR) model using Cauchy–Born rule. The simulations have been done based on two-scale method and equivalent fiber technique is used to decrease the amount of computation, by transferring the mechanical properties from nano- to microscale. In the model, the EF is dispersed in the RVE randomly, and Young’s modulus of nanocomposites has been extracted from simulations. Also the effect of clustering of CNTs in the matrix is investigated. Both experimental tensile test and Halpin–Tsai equation are used to compare and validate the results of simulation.

The results from this study illustrate that:

1. The PPR model is reliable for the modeling of interface in nanocomposites.
2. The results of modeling at the nanoscale showed that the EF length and the matrix thickness has a diminishing effect on elastic behavior of EF for EF length more than 300 nm. Due to the limitations in simulation of RVE, the minimum length of EF is considered 300 nm.
3. The results from this study have the proximity to experimental results and they predict more accurately than the calculated ones by Halpin–Tsai equation, especially at higher CNT loading.
4. Although some methods were used to disperse CNTs in the production of specimens, the increase in volume fraction has increased the differences between the numerical and experimental results. However, the maximum difference in Young’s modulus from the full dispersion model relative to experiment at the volume fraction of 1% is two-third lower than that between Halpin–Tsai equation and experiments.
5. The clustering of equivalent fibers increases the heterogeneity of the RVE, and by increasing the interaction between the equivalent fibers, their contribution to interacting with the environment is low, thereby reducing the properties of the nanocomposite.
6. The agglomeration of CNT in the nanocomposite degrades its effective elastic properties and the effective elastic properties decrease with the increase of the CNT agglomerate size.

## Figures and Tables

**Table 1:** The values of input parameters to the Park, Paulino, and Roesler (PPR) model.

Value	Input Parameter
$\begin{array}{l} \phi_{n}=0.2916081e^{-7}\\ \phi_{t}=0.1241959e^{-7} \end{array}$	Fracture energy (N/nm)
$\begin{array}{l} \sigma_{\max}=1.38861e^{-8}\\ \tau_{\max}=0.591409e^{-8} \end{array}$	Cohesive strength (N/nm²)
*α* = 5, *β* = 5	Shape parameters
*λ_n_* = 0.0851, *λ_t_* = 0.23516	Initial slope indicators

**Table 2:** Mechanical properties of multiwalled carbon nanotube [49,50].

Transversely Isotropic	
Young’s modulus (*E_z_*)	1 TPa
Young’s modulus (*E_x_* = *E_y_*)	30 GPa
Shear modulus (*G_xy_* = *G_xz_* = *G_yz_*)	30 GPa
Poisson’s ratio (*ν_xy_* = *ν_xz_* = *ν_yz_*)	0.0687
Density (*ρ*)	2.1 g/cm³

**Table 3:** Ratios of materials used for the production of nanocomposites with different volume fractions of multiwalled carbon nanotubes (MWCNTs).

Acetone (mL)	MWCNT (mg)	Hardener (g)	Epoxy (g)	CNT Vf.%	CNT wt.%
30	30	1.5	15	0.1	0.18
60	60	1.5	15	0.2	0.36
150	150	1.5	15	0.5	0.9
300	300	1.5	15	1	1.8

Volume fraction % = Vf.%; Weight fraction% = wt.%.

**Figure A1:**
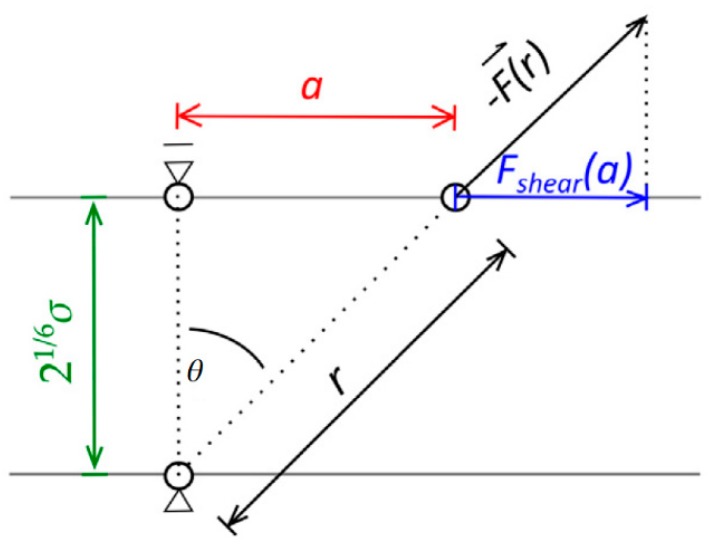
Tangential force component in atomic separation.

## Appendix A

To use three-dimensional PPR model in addition to normal interface behavior, shear behavior should be determined at this surface. Figure A1 shows how to calculate the tangential component of the van der Waals forces. By using the Equations (A1)–(A3) and substituting them in Equation (11), the tangential force at the interface is obtained using the Lennard–Jones (L–J) Potential, as shown in Equation (A4).

(A1) $$F_{shear}=F_{r}\cos\theta$$

(A2) $$\theta =\cos^{-1}\left(\frac{\sqrt[{6}]{\sigma }}{r}\right)$$

(A3) $$r^{2}=a^{2}+(\sqrt[{6}]{2}\sigma )$$

(A4) $$F_{shear}(a)=-4\frac{\varepsilon a}{a^{2}+2^{1/3}\sigma^{2}}\left[12\frac{\sigma^{12}}{{\left(a^{2}+2^{1/3}\sigma^{2}\right)}^{6}}-6\frac{\sigma^{6}}{{\left(a^{2}+2^{1/3}\sigma^{2}\right)}^{3}}\right]$$

## Appendix B

The Equation (A5) shows how to calculate the Young’s modulus of the homogeneous fiber nanocomposite based on the Halpin–Tsai model. The parameters *E*_11_ and *E*_22_ were calculated by the Equations (A6) and (A7). In these equations, *m* is related to matrix and the index *f* is related to the fiber. *E_L_* and *E_T_* are the longitudinal and transverse reinforcement Young’s modulus.

(A5) $$E_{c}=\frac{3}{8}E_{11}+\frac{5}{8}E_{22}$$

(A6) $$E_{11}=E_{m}\left(\frac{1+\frac{L}{R_{CNT}}\eta_{1}\upsilon_{f}}{1-\eta_{1}\upsilon_{f}}\right)$$

(A7) $$E_{22}=E_{m}\left(\frac{1+2\eta_{2}\upsilon_{f}}{1-\eta_{2}\upsilon_{f}}\right)$$

(A8) $$\eta_{1}=\frac{\frac{E_{longitudinal}}{E_{m}}-1}{\frac{E_{longitudinal}}{E_{m}}+\frac{L}{R_{CNT}}}$$

(A9) $$\eta_{2}=\frac{\frac{E_{Transverse}}{E_{m}}-1}{\frac{E_{Transverse}}{E_{m}}+\frac{L}{R_{CNT}}}$$
